# Industrial Control under Non-Ideal Measurements: Data-Based Signal Processing as an Alternative to Controller Retuning

**DOI:** 10.3390/s21041237

**Published:** 2021-02-10

**Authors:** Ivan Pisa, Antoni Morell, Ramón Vilanova, Jose Lopez Vicario

**Affiliations:** 1 Wireless Information Networking (WIN) Group, Escola d’Enginyeria, Universitat Autònoma de Barcelona, 08193 Bellaterra, Spain; antoni.morell@uab.cat (A.M.); jose.vicario@uab.cat (J.L.V.); 2 Advanced Systems for Automation and Control (ASAC) Group, Escola d’Enginyeria, Universitat Autònoma de Barcelona, 08193 Bellaterra, Spain; ramon.vilanova@uab.cat

**Keywords:** artificial neural networks, data-driven methods, denoising autoencoders, industrial control, wastewater treatment plants

## Abstract

Industrial environments are characterised by the non-lineal and highly complex processes they perform. Different control strategies are considered to assure that these processes are correctly performed. Nevertheless, these strategies are sensible to noise-corrupted and delayed measurements. For that reason, denoising techniques and delay correction methodologies should be considered but, most of these techniques require a complex design and optimisation process as a function of the scenario where they are applied. To alleviate this, a complete data-based approach devoted to denoising and correcting the delay of measurements is proposed here with a two-fold objective: simplify the solution design process and achieve its decoupling from the considered control strategy as well as from the scenario. Here it corresponds to a Wastewater Treatment Plant (WWTP). However, the proposed solution can be adopted at any industrial environment since neither an optimization nor a design focused on the scenario is required, only pairs of input and output data. Results show that a minimum Root Mean Squared Error (*RMSE*) improvement of a 63.87% is achieved when the new proposed data-based denoising approach is considered. In addition, the whole system performance show that similar and even better results are obtained when compared to scenario-optimised methodologies.

## 1. Introduction

Industrial systems are characterised by the highly complex and non-linear processes they require. These processes have to be performed under certain working conditions that have to be maintained over the time in order to assure a correct operation of the industrial plant. To assure this, different control strategies have been proposed, from the most simple ones, Proportional Integral and Derivative Controllers (PID), to the most complex model-based approaches like a mix between Model Predictive Controllers (MPC) and Fuzzy Logic Controllers (FLC) [1] (Chapter 1).

In that sense, two industrial fields where the control systems have been widely adopted and developed over the last years are the petrochemical industries and the wastewater facilities. Proportional Integral (PI) and Proportional Integral Derivative (PID) [2] controllers have been considered in [3,4,5]. In [3], two PI controllers have been proposed in a Wastewater Treatment Plant (WWTP) to control the dissolved oxygen in the fifth reactor tank ($S_{O,5}$) and the nitrite-nitrogen in the second one ($S_{NO,2}$). In [4], an hybrid controller based on Proportional Integral Derivative (PID) Controllers and FLC is proposed to control the temperature of a stripper devoted to producing gasoline and liquefied petroleum gas products, whereas in [6] an application approach is presented. On the other hand, in [5], two Fuzzy PID controllers are adopted to control a distillation column guaranteeing high control performance. However, in some cases more complex control structures are required. For instance, in [7] a unique MPC approach is considered to maintain the WWTP dissolved oxygen and the nitrite-nitrogen concentrations whereas in [8] MPCs have been considered to control several set-points of a depropanizer system. Hybrid control approaches have also been considered in different works like in [9], where MPCs complemented with FLCs are proposed to avoid violations of the effluent concentrations, understanding a violation as an exceed of the WWTP effluent limits. All these model-based controllers have a common point: they are based on mathematical models able to describe the behaviour of the processes to be controlled. However, the design and tuning procedures of such a kind of control strategies require a linearisation of the highly complex and non-linear mathematical models [1] (Section 2.7). This entails a degradation in the modelling of the processes under control. In that sense, Artificial Neural Networks (ANNs) have arisen as tools able to model highly complex and non-linear relationships without the necessity of a linearisation. They only require pairs of input and output data of the process being controlled [10] (Section 1). Not only this, the digital transformation and the Industry 4.0 paradigm is motivating the adoption of data-driven methods and ANNs in the modelling and control processes [11,12,13].

Data-driven methods and ANNs have arisen as new approaches able to offer a good control performance at the same time they increase the scalability and decoupling of the control strategy from the highly complex mathematical models [14,15,16,17,18,19]. In such a context, ANNs have been considered to perform different tasks: (i) act as soft-sensors, (ii) complement the model-based controllers, and (iii) act as a control strategy as such. ANNs acting as soft-sensors are able to measure certain components where a hardware-based sensors is either too expensive, or it has not been implemented yet. This is motivated by the ANNs ability in the modelling of highly complex and non-linear processes as well as by their easy tuning process [10] (Chapter 1). For instance, in [20], soft-sensors based on ANNs are proposed to determine the Chemical Oxygen Demand (COD), the Total Nitrogen (TN) and the Total Suspended Solids ($T_{SS}$) concentrations in the WWTP reactor tanks. Another approach is shown in [21], where Long Short-Term Memory (LSTM) cells have been considered to develop two soft-sensors predicting the ammonium and total nitrogen in the WWTP effluent. In terms of ANNs complementing the control strategies, neural networks have been considered in [22] to complement a MPC controller. There, three different non-lineal autoregressive exogenous model neural networks are in charge of determining the optimal set-points considered by an MPC-based control strategy devoted to controlling the ($S_{O,5}$) and the ammonium ($S_{NH,5}$) in the fifth WWTP reactor tank. Finally, ANNs have been considered in some works as the main control structure. In [23], an ANN-based Fuzzy Logic Controller is proposed to track the optimal set-points of the $S_{NO,2}$ and $S_{O,5}$ concentrations. In [24], ANNs model the direct relationships of an oil well drilling process. Later, they have been adopted either to determine the actuation variable of the control process, or to determine the next state of the system before being considered by a controller. Another example corresponds to [25], where two LSTM networks have been proposed to control the $S_{O,5}$ concentration by means of an Internal Model Controller structure. However, these nets have been designed to track a variable set-point. Other approaches correspond to Reinforcement Learning methodologies, where ANNs are considered as the main core of the reinforcement agents. One example where Reinforcement Learning is considered in the control of a WWTP corresponds to [26]. Here, the Reinforcement Agent has been trained observing the plant operators behaviour during a year.

In such a context, one of the common points among model-based controllers and the ANN-based ones is that they are sensible to perturbations of the signals involved in the control process [3,25]. The main perturbations that affect these signals are the noise and delays introduced by the different non-ideal sensors placed over the industrial plants. The appearance of time-delays is inevitable and as a consequence, they have to be corrected in order to avoid undesirable phenomena like undesired oscillations and the eventual instability of the control system [27]. In terms of the noise, the control strategies are characterised by the requirement of accurate control parameters and therefore, by the adoption of an accurate identification process. Thus, when noise is present in the measurements, the identification and proper controller tuning becomes a challenging task [28]. Besides, when noise is transferred to the manipulated variable, it may be easily amplified by the controller. This may originate the tear and wear of actuation mechanical parts, therefore degrading the performance and lowering instrumentation life. One clear example of time-delays and noise effects can be observed in the Benchmark Simulation Model No.1 (BSM1), a general purpose model of a WWTP plant [29], where the noise introduced by the considered sensors produces an incorrect measurement of the controlled variable and therefore, an incorrect actuation signal. On the other hand, the delays introduced by the same sensors entail a degradation of the control actuation. As a consequence, the reduction of the gain of the controller is required not only to reduce the delay effect and its propagation through time, but also to avoid the instability of the control system [30]. To alleviate these effects, different denoising and delay correction approaches can be considered: (i) from the denoising filter-based solutions [31,32] to the data-based denoising techniques such as Principal Component Analysis [33] and Denoising Autoencoders (DAE) [34], and (ii) from forecasting algorithms and controllers to the application of ANNs [35]. In [36], a mix between optimised, highly tuned and data-based denoising approaches is proposed. It implements two stages, one to denoise the measurements and the other to correct the introduced delays. The denoising process is performed adopting highly tuned low-pass filters whereas the delay correction process is performed by means of ANNs. This entails the increment of the controller’s gain and therefore, the control enhancement. The main drawback there is that the complete structure has been designed and optimised to offer a good performance in a specific scenario: its filters have been designed considering the type of signals as well as the type of sensors, whereas the ANNs correcting the delays are considering input and output pairs of data obtained from controlled structures. Thus, a high knowledge of the processes being controlled is required at the same time that the control solution will not be generalisable. This could be approached with the denoising and control approaches proposed in [25], where an ANN-based Internal Model Controller has been proposed to control the dissolved oxygen in the fifth reactor tank of the WWTP plant. Realistic sensors have been considered and as a consequence, the cleaning of noise corrupted measurements has also been proposed. However, this work neither considers the control of a fixed set-point, nor the correction of delays introduced by sensors.

For that reason, the approach proposed in [36] has been enhanced in this work by means of a complete and easy tuning data-based enhanced control solution. The new proposed approach will achieve the reduction of the design process complexity and the increment of its scalability [14,16]. It considers a new denoising approach, the data-based Denoising Stage. It is based on the application of ANNs whose main objective is to denoise the measurements involved in the control considering ideal and noise-corrupted measurements. In addition, this proposal also considers a delay correction stage, the ANN-based Delay Correction. It will correct all the delays affecting the control and actuation signals. Thus, the gain of the controller can be increased and consequently its actuation enhanced. The novelty here is placed in the ANNs training process. They will be trained considering input and output data obtained from open-loop configurations. Therefore, the decoupling of the proposal from mathematical models and specific controllers will be achieved. In other words, the proposed data-based Enhanced Control Strategy will correct the noise-corrupted and delayed measurements by means of the experience obtained from input and output data, neither requiring a highly complex tuning process, nor a deep knowledge of the processes being controlled.

Thus, the main contributions of this work can be summarised as:

- A complete data-based solution is proposed to improve an existing control strategy. Its main objectives are the reduction of the design complexity as well as the increment of the solution scalability.
- A deep knowledge of the process under control is not required since the proposed solution only requires input and output data to learn how to enhance the control system.
- Data-based methodologies and specially ANNs are considered as the main tools in the denoising process of measurements involved in the control process.
- ANNs in charge of correcting the delays introduced by the sensors and actuators will be trained with open-loop input and output data to assure the decoupling of the solution from the control topology.
- Results show that a similar and even better performance can be achieved when our approach is adopted instead of an optimized PI controller designed to work in a specific scenario. In our case, similar results are obtained when the control of a fix set-point is considered while the best performance is given when a variable set-point is tracked.

The structure of the paper is as follows: in Section 2 the material and methods adopted in this work are shown. Specially, the BSM1 framework and the DAEs are defined here. In Section 3, the data-based control approach with the proposed data-based Denoising and the ANN-based Delay Correction stages are described. In Section 4, the results in terms of the denoising performance, the delay correction and the whole structure behaviour are presented. Finally, Section 5 concludes the paper.

## 2. Material and Methods

### 2.1. Benchmark Simulation Model No.1

The proposed data-based Enhanced Control System will be implemented over a WWTP scenario consisting in a general purpose WWTP facility whose main aim is to manage the residual urban waters. Since WWTP facilities are critic infrastructures where any change or error in its behaviour can be translated into the pollution of the environment where the WWTP is placed, this work considers the adoption of the Benchmark Simulation Model No.1 (BSM1). It consists in a well-known and widely adopted digital framework modelling the behaviour of a general purpose WWTP [29]. BSM1 implements the Activated Sludge Model No.1 (ASM1) mathematical models which describe the biological and biochemical processes required to reduce the nitrogen derived pollutant components present in the incoming residual waters [37]. It is worth to remember that one of BSM1 objectives is to offer generality, easy comparison and results replication of different control strategies. For that reason, any new control strategy can be designed and tested in BSM1 before being implemented in a real scenario.

In terms of the pollutant components, incoming waters do not only contain nitrogen components, but also phosphorus ones. In such a context, the International Water Association (IWA) has also developed the Activated Sludge Models No.2 (ASM2), No. 2d (ASM2d) and No.3 (ASM3), which are able to model all the processes performed in the reduction of these other pollutants [38]. These models require more complex structures and frameworks such as the phosphorus removal BSM1 (BSM1-P) [39] or the Benchmark Simulation Model No.2 (BSM2) [40]. However, we have considered the adoption of BSM1 due to its simplicity [29]. Besides, the study of the chemical processes performed in the WWTP as well as its sludge processing are out of the scope of this work. Here we will only focus on the improvements provided by a data-based control strategy which can be implemented not only in WWTP environments, but also in any industry implementing a control strategy.

#### 2.1.1. BSM1 Architecture

**Figure 1:**
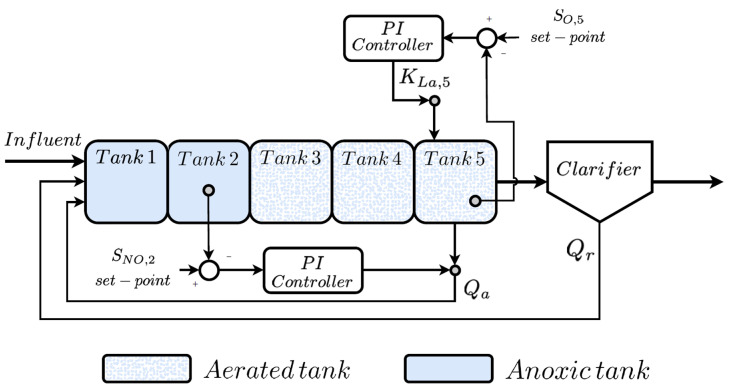
Benchmark Simulation Model No. 1 (BSM1) architecture. Notice that the first two reactor tanks are anoxic (working without oxygen) whereas the remaining three are working under aerated conditions.

BSM1 architecture can be observed in Figure 1. It considers five reactor tanks devoted to managing an average influent flow of 18,446 m${}^{3}$/day [29]. In addition, its total volume corresponds to 12,000 m${}^{3}$ divided in the following way: each one of the first two reactor tanks (anoxic tanks) have a total volume of 1000 m${}^{3}$, 1333 m${}^{3}$ the three aerated tanks and 6000 m${}^{3}$ the secondary clarifier. As a consequence, the total retention time, i.e., the time that the influent water lasts until it is spilled to its natural cycle, is equal to 14.4 h [29].

BSM1 influent considers a total amount of 15 variables which define the concentration of certain chemical components, the flow rate between tanks or the amount of solids in suspension inside a WWTP reactor tank [3,29]. These variables are also characterised by their high correlation in time. For that reason, they can be treated as time-series signals sampled every 15 min, the sampling time of the BSM1 model [29]. As stated before, BSM1 is a digital model of a general purpose WWTP. Consequently, the evolution of these 15 variables can be observed at any point of the plant. For instance, after the first two anoxic tanks, one can observe the effects of the nitrification process, where the nitrate components are transformed into nitrogen gas ($N_{2}$) and its derivates ($NO_{2}$). The denitrification process can be observed after the last three aerated tanks, where the concentration of ammonia present in the remaining water ($S_{NH}$) is reduced [41]. In such a context, nitrification and denitrification processes are correctly performed if certain components of the reactor tanks are in a determined range [41]. To achieve this, BSM1 framework incorporates two simple control strategies based on Proportional Integral controllers (PI) [3,42]. They are devoted to maintaining the nitrate-nitrogen concentration in the second reactor tank ($S_{NO,2}$) and the dissolved oxygen in the fifth reactor tank ($S_{O,5}$) at the desired set-points of 1 mg/L and 2 mg/L, respectively [3]. The first PI assures the $S_{NO,2}$ concentration modifying the internal ($Q_{a}$) and the external recirculation flows ($Q_{r}$) accordingly to the output of the PI controller. The PI in charge of the dissolved oxygen loop, compares the $S_{O,5}$ concentration with the give set-point in order to increase or decrease the oxygen transfer coefficient ($K_{La,5}$) [3]. This coefficient is directly related to the opening or closing of the fifth reactor tank oxygen valve.

#### 2.1.2. BSM1 Simulation Protocol

BSM1 implements a set of sensors able to measure the concentrations involved in the processes performed in the reactor tanks. They have two possible configurations: (i) an ideal one where all the measurements are gathered under ideal conditions neglecting the noise and delays introduced by the sensor itself, and (ii) a real analysis where the effects of non-idealities (noise and delays) are considered in the simulation of BSM1 and therefore, in the performance of the WWTP [3,29]. The model of noise considered by each sensor corresponds to the Additive White Gaussian Noise (AWGN) model, i.e., white zero mean an unit variance Gaussian noise. However, the variance is multiplied by the noise level defined in the sensor description, which equals to a 2.5% of the maximum value sensed by each sensor [29]. Among the different classes of sensors defined in [29], only two of them are considered here: the *A* and the $B_{0}$ sensor classes. This is motivated by the fact that BSM1 model recommends their adoption as the sensors to be considered to measure the different variables (see Table 14 in [3]). When working under realistic conditions, their behaviour can be described in terms of their transfer functions. As suggested in [3], *A* class sensors have a response time ($t_{r}$) which equals to one minute while $B_{0}$ sensors rising time is equivalent to 10 min. Their respective transfer functions are:

(1) $$G_{s}=\frac{1}{{\left(1+\tau s\right)}^{2}}$$

for the *A* class sensors, and:

(2) $$G_{s}=\frac{1}{{\left(1+\tau s\right)}^{8}}$$

for $B_{0}$ sensors. τ corresponds to the time constant of the transfer function. In the case of *A* class sensors it equals to $t_{r}/3.89$, where $t_{r}$ is equivalent to one minute. In terms of $B_{0}$ these variables correspond to $t_{r}/11.7724$ and 10 min for the τ and $t_{r}$ variables, respectively. For more details about the implementation of the considered sensors, readers are referred to [3] (Section 7).

In such a context, from the 15 variables of the BSM1 model this work only considers the following ones: the dissolved oxygen ($S_{O,4}$), the nitrate nitrogen ($S_{NO,4}$), the ammonium ($S_{NH,4}$), the flow rate ($Q_{4}$), the Total Suspended Solids ($T_{SS,4}$), all of them measured at the fourth reactor tank, the Total Suspended Solids in the input ($T_{SS,in}$), the $K_{La,5}$ and the $S_{O,5}$. $S_{NH,4}$, $Q_{4}$ and $T_{SS,4}$ have been considered because these variables are involved in the mass balance equation of the $S_{O,5}$ concentration, its conversion rate and the biological processes described in [36,37]. In addition, $S_{O,4}$ and $S_{NO,4}$ have also been considered due to the fact that they are two of the variables showing the highest mutual information with respect to $S_{O,5}$ (see Figure 5 in [25]). For more details about the selection of input variables taking into account the mutual information readers are referred to [43]. Finally, $S_{O,5}$ and $K_{La,5}$ are the variables involved in the control process. The configuration of their respective sensors can be observed in Table 1. It is noteworthy to mention that $K_{La,5}$ is not a measurable parameter, but an actuation value. In other words, it determines the opening and closing of the actuator which introduces a delay equal to four minutes [3].

In order to assure a fair comparison, BSM1 implements its own simulation protocol, which consists in the simulation of 14 days of daily influent variations. Depending on these variations, the different control strategies can be tested under different weather conditions. For that purpose, BSM1 implements its own influent profiles as a function of the weather: the dry, rainy and stormy influent profiles [40,44]. They can be defined as follows,

- Dry weather consists in 14 days without rainy episodes where daily variations are produced.
- Rainy weather consists in 14 days with a large rainy episode during days 9 and 10. Daily variations are still produced.
- Stormy weather consists in 14 days with two stormy episodes at days 8 and 11. Daily variations are still produced.

Regardless the weather condition, BSM1 model has to be carried out to its steady-state point before simulating a specific weather condition. This is performed by means of the initialisation process which consists in the simulation of a 100-days constant influent profile.

#### 2.1.3. Evaluation

As a framework offering generality, easy comparison, and replicable results, BSM1 adopts different metrics to determine the behaviour and the improvement achieved by the control strategies under analysis. These metrics are divided into two different groups: (i) environmental metrics, and (ii) control metrics [29,40]. Here, we will focus on the second ones since the main objective of this work is to offer a complete data-based control strategy which is independent and decoupled from the kind of industry where it will be applied. These metrics are the Integrated Absolute Error (*IAE*) and the Integrated Squared Error (*ISE*) which are measured only from the 7th day until the 14th one [29]. They are computed as follows:

(3) $$IAE=\sum_{n=7\mathrm{th}\;\mathrm{day}}^{14\mathrm{th}\;\mathrm{day}}\left|e\left[n\right]\right|\;ISE=\sum_{n=7\mathrm{th}\;\mathrm{day}}^{14\mathrm{th}\;\mathrm{day}}{\left(e\left[n\right]\right)}^{2}$$

where e[n]=r[n]−y[n] is the error between the reference signal or set-point and the feedback signal. In this case, if the dissolved oxygen control loop is considered, the reference signal equals to the $S_{O,5}$ set-point while the feedback one equals to its real measurement. In addition, these two metrics will be complemented for comparison purposes with the metrics defined in [36]: the average of the absolute error (mean(|e|)), its maximum (max(|e|)) and variance (var(|e|)) values. They are computed as:

(4) $$mean\left(|e|\right)=\frac{\sum_{n=7\mathrm{th}\;\mathrm{day}}^{14\mathrm{th}\;\mathrm{day}}\left|e\left[n\right]\right|}{N}$$

(5) max(|e|)=max(|e[n]|)

(6) $$var\left(|e|\right)=\frac{\sum_{7\mathrm{th}\;\mathrm{day}}^{14\mathrm{th}\;\mathrm{day}}{\left(e\left[n\right]\right)}^{2}}{N}-mean\left(|e|\right)$$

where *N* is the number of samples gathered from the 7th day of the simulation until the 14th day.

### 2.2. Data-Based Denoising Methods

The data-based Enhanced Control Strategy proposed in this work deals with industrial scenarios where the measurements involved in the control processes are corrupted by noise. To alleviate this, data-based denoising methods are adopted to obtain clean measurements from noise-corrupted ones. In that sense, denoising methods consist in a set of actuations devoted to reducing the effects of noise-corrupted measurements or signals. They have been applied in a great variety of fields, for instance, in the image or the signal processing ones [31,32]. Some of these methods consist in the application of low band-pass filters, wavelet transform and the application of certain estimations like the ones based on Least Means Squares [32,45]. However, data-based denoising techniques are mainly based on the application of denoising methods which only consider the data gathered from the scenario. This is the case for example of the Principal Component Analysis (PCA) considered as a denoising technique. The noise-corrupted signals are decomposed into two spaces: the signal and the noise space. Then, the signal is recovered only considering the signal space [33]. However, one of the main drawbacks in PCA is that offers its best performance when dealing with linear systems [46] (Chapter 13). To alleviate this, the Denoising Autoencoder (DAE), which can be interpreted as a non-linear extension of PCA, has been conceived [46] (Chapter 14).

**Figure 2:**
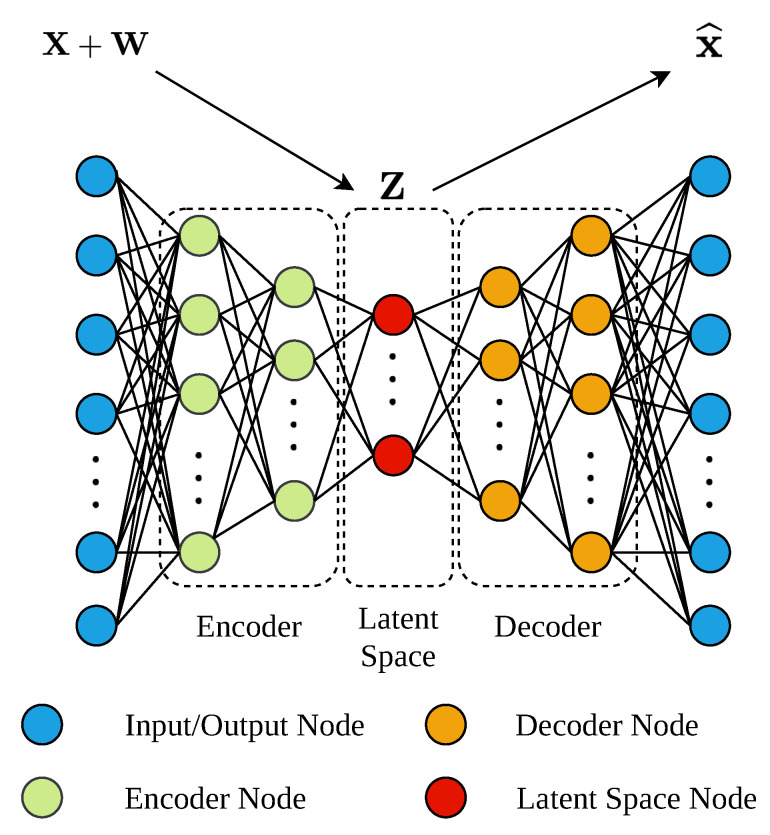
Denoising Autoencoder (DAE) structure. x and $\hat{x}\in R^{m\times 1}$ are the input and denoised data, respectively. $w\in R^{m\times 1}$ corresponds to the noise added by the different sensors. $z\in R^{k\times 1}$ vector corresponds to the data mapped into a latent space with k dimensionality.

The DAE consists in a neural network whose main objective is to transform the noise-corrupted measurements into clean ones [46] (Chapter 14). It has two clearly differentiated parts: (i) the encoder and (ii) the decoder. The encoder is in charge of taking the *m* noise-corrupted measurements and map them into a latent space of dimensionality *k*, which not only extracts the characteristics of the input measurements, but also reduces their input dimensionality (m>k). The decoder part performs the inverse action, it takes the outputs of the latent space and transform them into clean measurements with the same dimensionality as the inputs [47]. The type of ANNs considered in the DAEs implementation depends on the type of measurements. In such a context, DAEs can be designed either considering Multilayer Perceptron (MLP) networks (see Figure 2) in those cases where the time correlation between measurements is not so important [10] (Chapter 5). In those cases where the time correlation is predominant, MLPs with a Sliding Window (SW) or LSTM nets can be considered [46] (Chapter 10).

### 2.3. Modelling

As previously mentioned, the proposed data-based enhanced control system will be deployed over the BSM1 framework. It will be in charge of cleaning and correcting the noise and delays added by the sensors considered in [29]. In such a context, three different tools have been considered to implement the proposed data-based control strategy. They correspond to Simulink, Matlab and Python. Simulink has been considered as the simulation environment where the data-based control strategy is implemented and tested. BSM1 is also implemented in this environment. The proposed data-driven control strategy considers a delay correction module, where the delays added by the different sensors can be corrected by means of ANNs. These ANNs have been implemented in Matlab (version R2020b) with the Neural Networks Toolbox and the Neural Net Fitting tool. The metrics considered in the BSM1 evaluation are also implemented in Matlab. Finally, the ANNs considered in the implementation of the data-based Denoising Methods have been designed considering Python and trained with a NVIDIA GeForce RTX 2080 Titan GPU to speed up the ANN training process. In this case, Python version 3.6 and the following open-source libraries have been adopted in the design of the ANNs:

- NumPy (1.18.1) [48]: NumPy library has been considered to manage the vectors and matrices involved in the ANNs training process.
- Pandas (1.0.3) [49]: Pandas is adopted to load the input and output data from Matlab and Simulink into Python environment.
- Scikit-Learn (0.22.1) [50]: Scikit-Learn library implements most of the functions considered in the data preprocessing techniques, cross-validation of results and evaluation processes. In this case, it has been considered to normalise and denormalise the ANNs input and output data, to divide the input data into training and test datasets and to perform the K-Fold cross-validation [51,52]. The metrics considered in the evaluation of the denoising autoencoders are obtained from this library.
- Tensorflow (1.14.0) [53]: The open-source Tensorflow machine learning framework and its Keras API have been considered to implement the different ANNs adopted in the DAE implementation. Since Keras API is adopted, the architectures considered here have been derived from predefined ANNs structures.

## 3. Data-Based Enhanced Control Strategy

One of the main objectives of this work is to design a data-based enhanced control strategy which can be deployed at any industry regardless its design and purpose. Measurements original quality is one of the key factors that determines the operational performance. Based on this, in this work the control enhancement will be directed towards a data-driven processing of the process measurements before it reaches the controller. The main purpose is to minimise the effect of noise and delays therefore allowing the controller to be as much transparent as possible to these effects. To achieve this, the proposed control enhancement strategy is mainly based on data obtained from the industrial scenario where it will be applied. In this case, the scenario considered corresponds to a WWTP where a PI-based control strategy was previously implemented [36]. That control strategy was devoted to managing the $S_{O,5}$ and maintaining it at the desired value of 2 mg/L. It considers two different stages in charge of denoising the measurements and correcting the delays of the different sensors. The denoising stage considers moving average low-pass filters, a classical denoising method which reduces the effect of the higher frequency components and therefore the effects of noise [31,32]. The second stage, i.e., the delay correction one, adopts ANNs to predict the level of $S_{O,5}$ ten minutes in advance with respect to the instant where measurements are obtained. As a consequence, the effects of possible delays added by the sensors as well as by the filter-based denoising approach can be mitigated. However, these ANNs have been trained considering data from scenarios with a closed-loop configuration or, in other words, where a control strategy is already working [36]. In addition, that control strategy has been designed and optimised to work under a certain scenario, the WWTP one. Thus, the proposed denoising and delay correction methods are designed considering specificities of the scenario and as a consequence, losing their generalisation and scalability.

**Figure 3:**
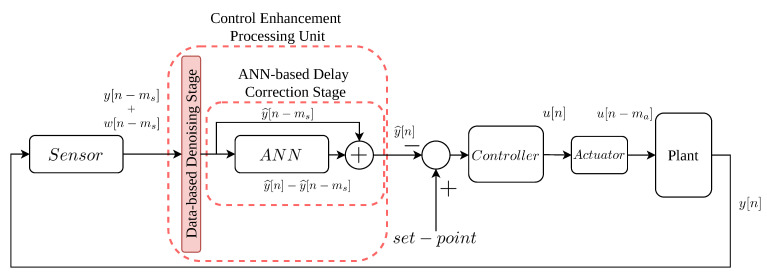
Data-based Control Structure. *u* is the actuation signal whereas $\hat{y}$ corresponds to the clean estimation of the controlled signal. It is also part of y, the vector of measurements obtained from the different sensors. $m_{s}$ and $m_{a}$ are the delays introduced by the sensors and the actuator, respectively.

Our proposal here is the data-based enhanced control approach shown in Figure 3. It can be understood as an improvement of the control strategy defined in [36]. However, our approach is mainly based on input and output data of the process being controlled. Therefore, it can be implemented without requiring a deep knowledge of the system where it is going to be deployed. Moreover, the fact that this approach is based only on data allows its application at any industrial environment since only input and output pairs of measurements of the processes being controlled are needed. These measurements can be obtained following two manners according to the industrial scenario. If the scenario is not being controlled, input and output measurements can be directly obtained from sensors and systems monitoring the processes performed in the industrial plants, for instance, the SCADA systems. In the case where the scenario is already being controlled, these pairs of measurements can be obtained from monitoring systems as well. However, the effects of the considered control structure will be intrinsically observed in the measurements. Therefore, the decoupling of the proposed approach from the control strategy will not be achieved. If the decoupling is desired, the industrial environment should be working in an open-loop configuration if it is available. Otherwise, a digital model or a digital twin of the industrial plant could be adopted to simulate its open-loop behaviour without interrupting or changing the industrial processes.

In this case, our proposal considers the complete control process from the sensor to the output of the actuator just before entering in the plant. In the case of a WWTP, the sensor will be in charge of determining the controlled, delayed and noise-corrupted variable ($y\left[n-m_{s}\right]+w\left[n-m_{s}\right]$), i.e., the $S_{O,5}$ measured. The novelty here is in the data-based Denoising Stage and the Delay Correction Stage (red blocks in Figure 3). Now, the denoising stage will be implemented considering DAEs and ANNs instead of classical denoising approaches like low-pass filters. As a consequence, the delays introduced by the low-pass filters can be neglected at the same time the denoising process is improved. However, the delays introduced by the real sensors ($m_{s}$) are still present in the measurements. They will be corrected in the ANN-based Delay Correction Stage.

As in [36], the ANNs will predict the difference between the delayed measurements and the real ones ($\hat{y}\left[n\right]-\hat{y}\left[n-m_{s}\right]$). Later, the delays added by sensors will be corrected obtaining an estimation of the ideal value with the correction of the actuator delay ($\hat{y}\left[n\right]$) at the output of the ANN-based Correction Stage. Then, this output will be compared to the given set-point and transformed into the corresponding actuation signal (u[n]). Notice that the actuator adds a delay equal to $m_{a}$, however, the forecasted measurements as well as the actuation signal given by the PI controller will consider this amount of time (the forecasting time considers the delays introduced by the sensors and the actuators). Thus, the actuation signal entering in the plant ($u[n-m_{a}]$) equals to a non-delayed actuation signal with respect to the forecasted control one ($\hat{y}\left[n\right]$). In this case, the ANN-based Delay Correction nets will be trained considering data from an open loop configuration of the process under control. Thus, the effects of the controller are not present in the training of the ANNs and therefore, achieving the decoupling of the control strategy from the controller type.

As a summary, our data-based enhanced control strategy, which is implemented by means of ANNs, allows us to improve a control approach by means of (i) decreasing the design process complexity, and (ii) increasing its scalability [14,16]. The complexity reduction is achieved due to the fact that ANNs do not require a such precise adjustment to the scenario as usual filtering strategies do. Besides, the scalability is increased since ANNs can be applied in different industrial scenarios (not constrained to WWTP ones): the knowledge of the controlled scenario will be directly derived by ANNs if they are trained with proper data [10] (Chapters 1, 2, 5 and 17). Results in Section 4 show that our data-based enhanced control approach is able to offer similar and even better results in the control performance than the ones obtained in [36], a control approach specially designed and optimised to work in WWTP scenarios.

It is worth to noting that the proposed data-based Enhanced Control system has been designed and implemented over the BSM1 framework. In a real scenario, the ANNs considered in the proposed strategy should be trained considering an offline training process to avoid the interruption or malfunctioning of the WWTP behaviour. The networks will be trained and tested outside the industrial plants considering the measurements obtained from the monitoring or SCADA system of the plant. Once trained and tested, they will be implemented as a complement of the WWTP monitoring system. In that sense, only the weights and biases of the ANNs are required to implement the whole structure since each ANN can be completely defined adopting its respective weights and biases.

### 3.1. Data-Based Denoising Stage

The data-based Denoising Stage is required to clean the measurements obtained from the real sensors considered in [29]. This is an important process due to the fact that the performance of the control strategy is directly related to the measurements quality. For instance, DAEs have been considered in [25], where the improvement achieved in the control performance is around a 16.84% in average with respect to the situation where DAEs are not adopted. Besides, the control performance is also dependant on the denoising quality. Again, in [25], the best denoising approach is able to improve the performance in six percentage points with respect to the worst denoising approach.

Among the different denoising methods available in the literature (PCA, Low-pass and Band-pass filters, Wavelet Transformations, Denoising Autoencoders, etc., [32,33,34,54]), we have adopted two MLP-based denoising structures due to their good performance when dealing with signals showing a high correlation in time [34,54]. Although recurrent LSTM networks have been designed to work with such time-correlated signals, we have considered MLP-based structures for two reasons: their complexity is reduced with respect to LSTM nets, and they also show a good performance when dealign with WWTP measurements [25]. Besides, the time correlation between measurements is still preserved by means of the Sliding Window (SW), which not only sorts the measurements in time, but also helps in the denoising process.

#### Denoising Architectures

**Figure 4:**
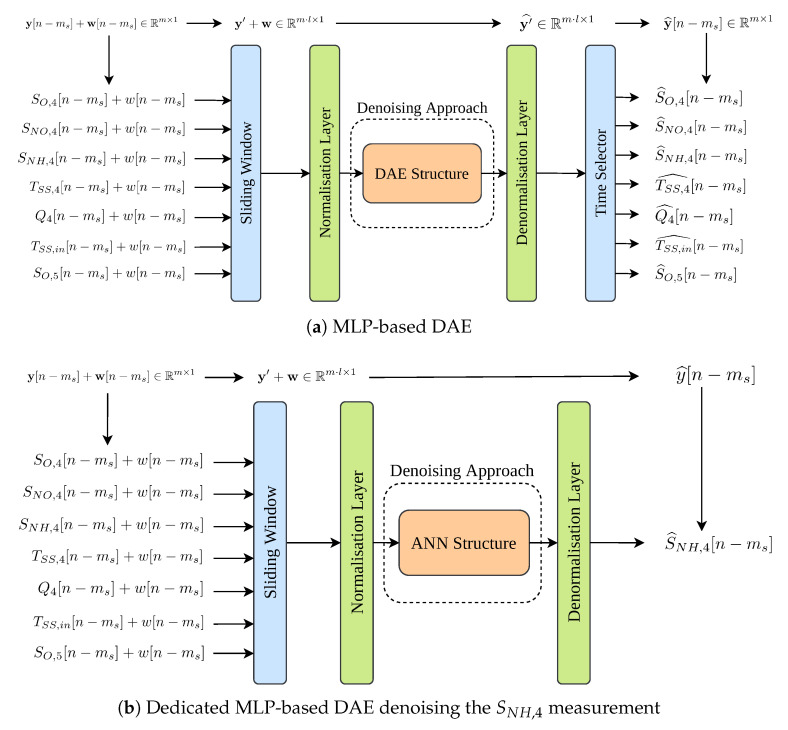
MLP-based Denoising Architecture. Notice that the main difference is placed after the denoising approach. The MLP-based DAE obtains a clean version of the measurements, i.e., a column vector of length m·l, where *m* represents the number of input measurements and *l* the length of the sliding window. The Dedicated MLP-based DAE directly estimates a clean version of a unique measurement.

A classical denoising method, a moving average low-pass filter, has been considered to clean the measurements involved in the control in [36]. It corresponds to a weighted moving average filter which gives more information to the new measurements to decrease the filter delay during long variations of the measured signal. In this case, the filter multiplies the average of the 25% of the new measurements by 0.7 whereas it multiplies by 0.3 the rest of the data [36]. In such a case, the filter has been able to reduce the noise effects at expense of adding extra delays. Not only this, the denoising performance can be still improved since it does not consider any kind of knowledge about the noise affecting the measurements. For that reason, two different MLP-based denoising architectures have been considered: (i) a MLP-based DAE, and (ii) a Dedicated MLP-based DAE, both considering a Sliding Window (SW) of 4 h (see Figure 4). The former corresponds to the structure shown in Figure 4a whereas the Dedicated MLP-based DAE approach is shown in Figure 4b. Both architectures have a common structure in charge of the data preprocessing tasks: the Sliding Window and the Normalisation Layers. Here, the Sliding Window Layer is adopted to sort the measurements in time and also to preserve the time-correlation between measurements. Its length has been obtained through the process explained in [55], where it is shown that the minimum periodicity of input variables should be considered as the minimum length of the SW, here the SW length has been set to 4 h. Then, the sorted measurements are normalised towards zero mean and unit variance in the Normalisation Layer, which is placed previously to the Denoising Approach. This layer is considered to address the heterogeneity of data since the different variables of BSM1 are widely heterogeneous as shown in the saturation values of BSM1 sensors (see Table 1).

**Figure 5:**
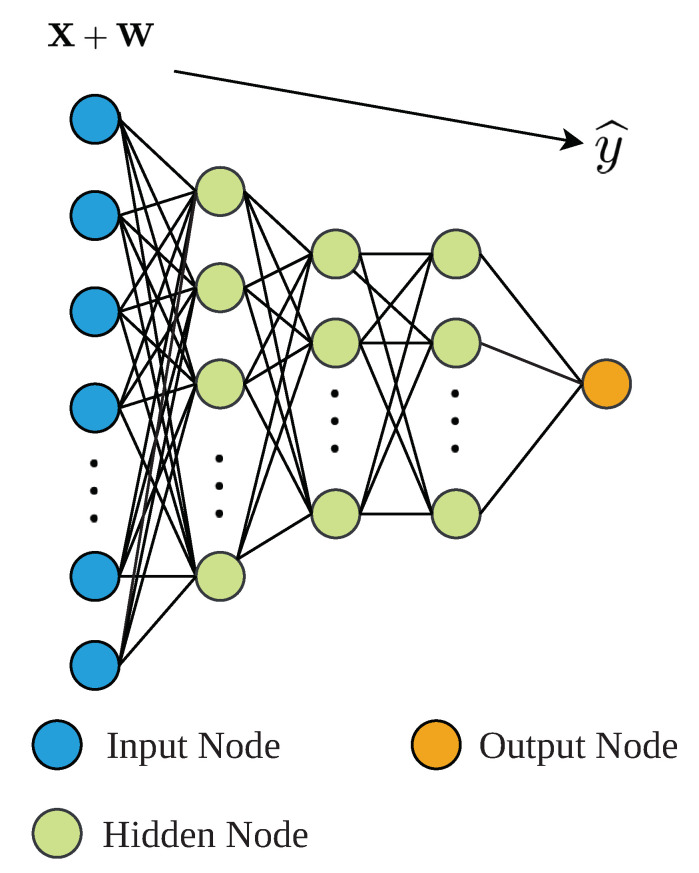
Dedicated MLP-based DAE denoising method. $\hat{y}\in R$ corresponds to one of the variables involved in the control strategy.

The denoising approaches of both architectures differ in the topology of the considered net in their implementation. The MLP-based DAE will take the vector of noise measurements sorted in time and normalised as the input data and will return a cleaned version of it (see Figure 2 and Figure 4a). The dimension of input and output vectors are exactly equal, $R^{m\cdot l\times 1}$, where *m* and *l* are the number of input variables and the length of the sliding window, respectively. However, the input vector has noisy measurements while the output one has the same measurements without noise. Then, the clean measurements are denormalised in the Denormalisation layer and finally, only the last cleaned measurements per variable are selected in the Time Selector layer. The denoising approach considered in the Dedicated MLP-based DAE structure (see Figure 4b) corresponds to a modified MLP-based DAE. It will directly obtain an estimation of the clean measurements of a unique variable instead of mapping the inputs into a latent space and then recover a clean version of them (see Figure 5). Then, this estimation is denormalised in the Denormalisation Layer.

In terms of the input measurements, both architectures consider inputs obtained from the mass balance equation of the $S_{O,5}$ variable, its conversion rate and the biological processes described in [36,37]. These variables are:

- $S_{NH,4}$ (mg/L): the ammonium concentration present at the output of the fourth reactor tank.
- $T_{SS,4}$ (mg/L): the total suspended solids at the output of the fourth reactor tank.
- $Q_{4}$ (m${}^{3}$/day): the flow rate at the output of the fourth reactor tank.
- $T_{SS,in}$ (mg/L): the total suspended solids at the input of the WWTP plant.
- $S_{O,5}$ (mg/L): the dissolved oxygen in the fifth reactor tank

Not only this, extra variables are considered to complement the MLP-based Denoising architectures. They correspond to the $S_{O,4}$ and the $S_{NO,4}$, both measured in (mg/L). They are two of the variables showing the highest mutual information with respect to the $S_{O,5}$ concentration [43,56,57].

The grid search methodology has been considered to determine the internal structure of both denoising approaches, i.e., their hyperparameters. This methodology has been considered as one of the most effective methods to determine the ANNs hyperparameters testing different net configurations [58,59]. Here, it has been considered to determine the number of hidden layers and hidden neurons per layer of the two denoising approaches. Once it finishes, different structures will be obtained. Consequently, the structure performing better is the one which should be considered in the neural network training process. After performing the grid search, two optimal structures will be obtained. Then, they will be trained and tested with new data in order to determine their denoising performance. The results of the grid search show that the best architectures are:

- MLP-based DAE: Denoising Autoencoder structure with two hidden layers as the encoder part, a hidden layer acting as the latent space and two hidden layers as the decoder. The two layers forming the encoder consider a total amount of 100 and 50 hidden neurons, respectively. The latent space considers 25 hidden neurons and the decoder layers consider 50 and 100 hidden neurons, respectively. Each hidden node implements a Rectified Linear Activation (ReLU) function with the exception of the last hidden layer which implements a Linear Activation function in each node [10] (Chapter 1).
- Dedicated MLP-based DAE: The Dedicated MLP-based DAE of the second denoising approach considers three hidden layers where the first one considers 100 hidden neurons whereas the last two 50 hidden nodes. Here, the last hidden layer corresponds to a unique node which implements a Linear Activation function. The rest of nodes consider a ReLU function.

In both cases, an initial learning rate of $1\times 10^{-3}$ and a total amount of 500 epochs have been considered to perform the grid search. It has been performed considering the Back-propagation and Adam training and optimisation algorithms [46] (Sections 6.5 and 8.5.3). As in most of the neural networks, regularisation techniques have been applied to avoid the overfitting problem, i.e., the memorisation of input and output data. Thus, applying them we assure the generalisation of the denoising approaches [46] (Chapter 7). To alleviate the overfitting problem, two different techniques have been considered: (i) L2 extra-penalty [46] (Section 7.1), and (ii) early stopping [46] (Section 7.8). In this context, the L2 parameter of the L2 extra-penalty and the patience of the early stopping technique (number of times that a worsening of the training metrics is allowed before ending up the training process) have been obtained after performing the cross-validation of the networks. It has been performed adopting the K-fold method with five folds [51]. The L2 parameter has been set to $1\times 10^{-3}$ for the MLP-based DAE structure and $1\times 10^{-4}$ for the Dedicated MLP-based DAE net. The early stopping patience is equal to 10.

The data considered in the design of the data-based Denosing Stage have been generated in the BSM1 framework simulating twice a whole year influent of the BSM1 framework. Besides, the sensors have been configured considering their two possible configurations. As a consequence, four datasets have been obtained: two considering ideal measurements and the other two considering the equivalent noise-corrupted and delayed measurements. One pair of datasets have been considered in the grid search while the others are considered in the cross-validation process. Finally, each datasets has been divided into the usual 70-15-15 percentage distribution, where the first 85% of data are considered for training purposes and the remaining 15% for testing ones.

Before deploying one of the two proposed structures, they have to be tested to decide which one is performing better. This is motivated by the pros and cons of each structure. The MLP-based DAE is implemented considering only a unique structure which will be able to denoise all the considered noise-corrupted measurements. However, its denoising efforts are divided among all the measurements. On the other hand, as many Dedicated MLP-based DAEs as sensors have to be implemented in order to denoise the measurements involved in the proposed approach. In this case, the number of required DAE nets is increased at expense of focusing their denoising efforts in a specific measurement. Thus, the trade-off between the number of required ANNs and their denoising performance has to be solved.

### 3.2. ANN-Based Delay Correction Stage

**Figure 6:**
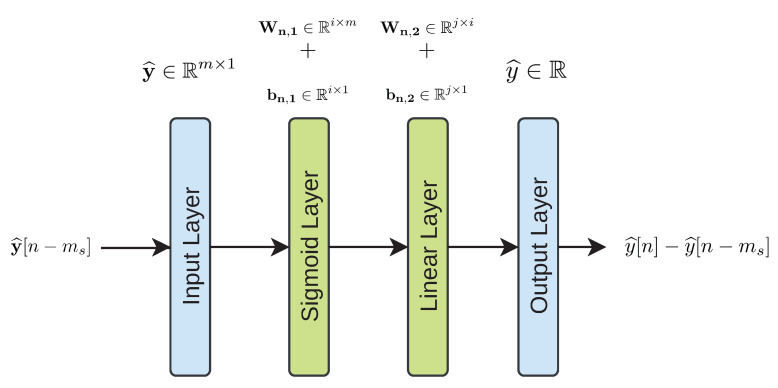
ANN-based Delay correction net. $\hat{y}$ corresponds to the vector of denoised measurements and $\hat{y}$ corresponds to the controlled variable. $b_{n,x}$ and $W_{n,x}$ are the biases and weights of the *x*th hidden layer, respectively. *m* is the number of variables considered in the ANN-based Delay Correction net. Hidden layers are green coloured while input and output layers are depicted in blue.

The Delay Correction Stage purpose is to correct the delay introduced not only by the sensors, but also by the actuator by means of predicting the controlled variable of the control strategy, i.e., the $S_{O,5}$. This is performed by means of a simple MLP network which has been designed with the Neural Net Fitting tool of Matlab’s Neural Network Toolbox in order to decrease the complexity of the ANN-based Delay Correction Stage. The MLP architecture is already defined by the same neural network fitting tool. It corresponds to a simple MLP feedforward network with two layers (see Figure 6), where the first one corresponds to a Sigmoid Layer (sigmoid activation function [10] (Chapter 1)) while the second one corresponds to a Linear Layer (linear activation function [10] (Chapter 1)). The sigmoid layer dimension *i* can de determined by the designer while the Linear Layer dimension is fixed to 1, j=1, by default.

Here, the input data correspond to *m* denoised and delayed measurements which will be modified by the weights and biases of the Sigmoid Layer, $W_{n,1}\in R^{i\times m}$ and $b_{n,1}\in R^{i\times 1}$, respectively. The number of hidden neurons in this layer, which can be defined by the designer, is equal to *i*. Then, the outputs of this hidden layer are modified by the Linear layer, whose weights and biases are $W_{n,2}\in R^{i\times j}$ and $b_{n,2}\in R^{j\times 1}$, respectively. Finally, the output of the net corresponds to a prediction of the difference between the expected values of the controlled signal with the actuator delay correction, $\hat{y}\left[n\right]$, and the delayed controlled signal $\hat{y}\left[n-m_{s}\right]$. This difference is computed because the ANNs trained in Matlab have a better performance when they predict this difference instead of directly predicting the delay-corrected $S_{O,5}$ measurements [36]. Therefore, the output of the whole ANN-based Delay Correction Stage (see Figure 3) will be equal to $\hat{y}\left[n\right]$. Then, the controller will compute the actuation variable accordingly to its input, u[n]. Finally, the signal obtained after the actuator, and therefore, the signal entering in the plant corresponds to $u[n-m_{a}]$, which is an estimation of the actuation signal derived from the ideal controlled variable. This signal corresponds to the oxygen transfer coefficient in the fifth tank, i.e., the $K_{La,5}$ [3].

The number of hidden neurons in the Sigmoid Layer of the MLP network has been set to 20, since results show that a good prediction performance is achieved. Here, the Bayesian regularisation algorithm is adopted [60] considering a maximum number of epochs equal to 1000. Moreover, the cost function to optimize in the training process corresponds to the Mean Squared Error (MSE). Input and output pairs of data have been split again in the following distribution: 70% for training purposes, 15% for validation purposes and 15% for testing ones.

The vector of input measurements has been determined considering different configurations of input variables. They have been selected accordingly to the mass balance equations of $S_{O,5}$, the type of control and the simulated weather profile as well. As a result, three different configurations have been tested for the Dry, Rainy and Stormy weathers when either a fixed set-point, or a variable one is considered in the control strategy. These configurations are:

- ANNconf1: It considers the $S_{NH,4}$, the $T_{SS,4}$, the $Q_{4}$, the $T_{SS,in}$, the $K_{La,5}$, the $S_{O,5}$ and a storm flag which is enabled when $T_{SS,in}$ is over 400 mg/L.
- ANNconf2: It considers the same inputs as ANNconf1, but the storm flag is changed by the readily biodegradable substrate in the fourth reactor tank (mg/L), which is measured with the software sensor proposed in [61].
- ANNconf3: It considers the same inputs as the ANN-based denoising architectures adding the actuation variable, the $K_{La,5}$.

**Figure 7:**
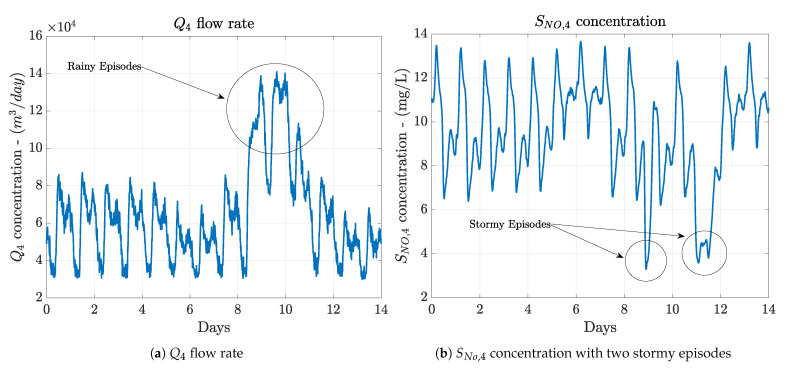
Variables where the effects of rainy and stormy episodes are more noticeable.

Among the different variables, $T_{SS,in}$ and $Q_{4}$ will be considered to detect the topology of weather since $Q_{4}$ values higher than $9.24\times 10^{4}$ m${}^{3}$/day will be observed when rainy and stormy events are produced. In that sense, $T_{SS,in}$ and $S_{NO,4}$ will determine when a stormy event is produced whenever $T_{SS,in}$ values are placed over 400 mg/L or $S_{NO,4}$ is below 5.5 mg/L (see Figure 7a,b).

Finally, each configuration has been trained considering a BSM1 open-loop configuration, i.e., without any kind of control. This is performed to achieve the decoupling the ANN-based Delay Correction stage from the considered controller. Moreover, this also decreases the design complexity of the whole structure since no controller is required. This entails that the ANN-based Denoising Stage can be designed at the same time as the controller. Thus the same data can be adopted. Otherwise, the controller has to be implemented and deployed before generating the data considered in the delay correction stage. In addition, for comparison purposes the different measurements have been obtained performing the same pattern of simulations as in [36]. This pattern corresponds to the simulation of 35 days of the WWTP behaviour. The first 21 days correspond to the simulation of a dry weather considering the following variations of $K_{La,5}$: seven days between 45 and 245 days${}^{-1}$, seven days between 5 and 355 day${}^{-1}$ instead, and seven days with a fixed $K_{La,5}$ equal to 145 day${}^{-1}$. The last 14 days correspond to the simulation of seven days of rainy and seven days of stormy weather profiles, respectively. Notice that ANNconf1 and ANNconf2 adopt the same input measurements or variables as the ones considered in ANN3 and ANN4 configurations of [36]. Nevertheless, the nets considered here have been trained considering only open-loop configurations.

## 4. Results

Results have been computed to determine the performance of those stages where a novelty has been proposed with respect to [36]: ANNs are considered in the design of the data-based Denoising and the ANN-based Delay Correction stages. The former considers ANNs instead of Moving Average Low-pass filters to denoise the noise-corrupted and delayed measurements. Thus, the proposed data-based Denoising stage will achieve a two-fold objective: (i) base the denoising strategy only on data, and (ii) simplify its design and implementation process since this solution does not require neither an exhaustive adjust of the filters to the characteristics of the controlled system, nor a deep knowledge of the scenario where it is implemented. The ANN-based Delay Correction stage, which has been firstly designed in [36], is now trained with data obtained from a non-controlled WWTP scenario (open-loop configuration) instead of a controlled one (closed-loop configuration). Applying this, we try to assure the decoupling of the delay correction from the considered control strategy. Results will show that the proposed system is able to yield similar and even better results than the system proposed in [36] where similar approaches have been designed and optimized towards the scenario.

Three different analysis of the WWTP performance have been carried out, one analysis to determine the behaviour of the data-based Denoising Stage, another to compute the performance of the ANN-based Delay Correction Stage, and the last one which shows the performance of the whole system. The data-based Denoising performance will be computed and analysed in terms of the Root Mean Squared Error (*RMSE*), the Mean Absolute Error (*MAE*), the Mean Average Percentage Error (*MAPE*) and the determination coefficient ($R^{2}$) since these are metrics commonly considered when dealing with ANNs [62]. All of them have been computed over the test dataset and considering normalised measurements with the exception of the *MAPE*, which considers denormalised data to avoid divisions by zero. The performance of the ANN-based Delay Correction stage will be given in terms of the metrics adopted in the ANN training process, i.e., the MSE and the $R^{2}$, whereas the whole system performance will be computed in terms of *IAE* and *ISE* as well as in terms of the aforementioned error derived ones (mean(|e|), var(|e|) and max(|e|)).

In that sense, the *RMSE* is computed as

(7) $$RMSE=\sqrt{\frac{1}{N}\sum_{n=1}^{N}{\left(y\left[n\right]-\hat{y}\left[n\right]\right)}^{2}}$$

where *N* corresponds to the number of samples being denoised, $\hat{y}$ to the denoised measurement and y[n] to the ideal value. *RMSE* gives an idea of the topology of errors present in the denoising stage since it penalises more the high errors than the lower ones. For that reason, *RMSE* metric is complemented with the *MAE* error, which indistinctly penalises the errors. It is computed as

(8) $$MAE=\frac{1}{N}\sum_{n=1}^{N}\left|y\left[n\right]-\hat{y}\left[n\right]\right|.$$

It can also be defined as an absolute metric. In other words, the *MAE* is not able to say if an error is too big or otherwise it is very low. For that reason *MAPE* has also been considered,

(9) $$MAPE=\frac{1}{N}\sum_{n=1}^{N}\frac{|y\left[n\right]-\hat{y}\left[n\right]|}{\left|y[n]\right|}.$$

*MAPE* corresponds to a metric which computes the percentage error with respect to the ideal value, therefore, telling how big is the error performed by the denoising approach. All these metrics compute the difference between the ideal and the denoised values. For that reason, the lower the values of the metrics, the better the denoising performance. Moreover, an extra metric, the $R^{2}$ has been adopted to determine the correlation between the denoised and the ideal measurements. Here, values closer to 1 are sought, since a $R^{2}$ equal to 1 is translated into a perfect correlation whilst $R^{2}$ equal to 0 means that there is no correlation between predicted and target values.

### 4.1. Data-Based Denoising Performance

The data-based Denoising performance is shown in Table 2, where the results of the proposed data-based denoising techniques are computed in terms of *RMSE*, *MAE*, *MAPE* and $R^{2}$. The classical denoising approach, i.e., the moving average low-pass filter denoising approach proposed in [36], is also considered as a baseline showing the minimum available performance. One of the most clear points is that data-based methodologies overcome the performance offered by the moving average low-pass filter in terms of the *RMSE* and the *MAE*. For instance, the MLP-based DAE improves the *RMSE* and the *MAE* a 63.87% and a 61.29%, respectively. These improvements are increased until a 87.32% and a 86.56% when the Dedicated MLP-based DAEs are considered. Results also show that the best denoising approach corresponds to the Dedicated MLP-based DAEs, which are able to offer an average *RMSE* equal to 0.033, an average *MAE* of 0.025, an average *MAPE* of 1.27% and a $R^{2}$ coefficient equal to 0.998.

In addition, although the MLP-based DAE is a similar approach to the Dedicated MLP-based DAEs and its performance shows low *RMSE* values, it has two critical points: the $S_{NH,4}$ and the $S_{O,5}$*MAPE* values equal to 11.14% and 5.18%, respectively. To determine their effects, we will show a simple example. Lets suppose that a real $S_{O,5}$ concentration equal to 2 mg/L is present in the WWTP fifth reactor tank. When measured, the value will be corrupted by noise and delayed by the sensor. To alleviate this, the MLP-based DAE is considered, however, the range where the $S_{O,5}$ denoised measurement will be placed corresponds to [1.90,2.10], which is a wide range and therefore, inaccurate. On the other hand, the Moving Average Low-pass Filter approach can be selected as well. Again, we will observe the same problem since its *MAPE* value is even higher (17.87%). The solution therefore is to consider the Dedicated MLP-based DAE, whose *MAPE* value is equal to 2.15%. Thus, the range where the denoised $S_{O,5}$ measurement can be placed is drastically reduced until the [1.96,2.04] range. This will entail that the controller will compare a more accurate $S_{O,5}$ measurement with the desired set-point. So, the lower the *MAPE*, the higher the accuracy, the better the denoising process and therefore, the better the control.

In terms of $R^{2}$ metric, all the approaches are able to show a good correlation between denoised and ideal measurements. The exception here is placed in the classical denoising method, the low-pass filter denoising the $T_{SS,4}$, even though it is able to offer a good *MAPE*.

From the point of view of the ANNs training process, the Dedicated MLP-based DAEs are able to offer such a good performance due to their architecture, where all the considered inputs are directly related to a unique output. In the case of the MLP-based DAE, the considered inputs are related to the same number of outputs. Consequently, the denoising approach has to divide its efforts in the denoising process of multiple variables instead of focusing them in cleaning a unique measurement. This is also clearly observed in terms of the Training Time. The MLP-based DAE requires a total amount of 113.53 s while the Dedicated MLP-based DAEs highest training time equals to 62.25 s. Nearly the half of the MLP-based DAE training time.

**Figure 8:**
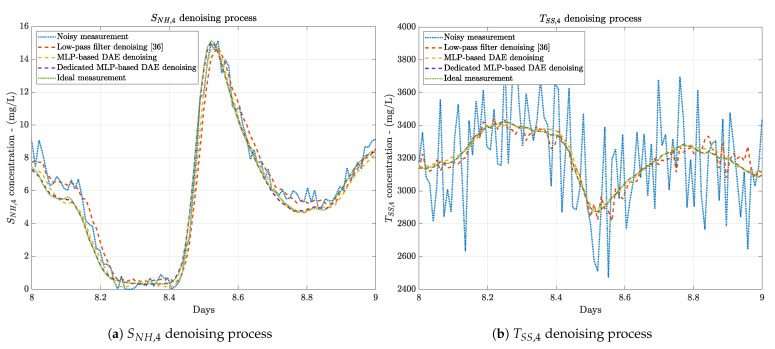
Denoising Process performed by the different approaches. Notice that in the $T_{SS,4}$ denoising process the Dedicated MLP-based DAE output is hidden by the Ideal output.

All these points motivate us to consider the Dedicated MLP-based DAEs as the denoising approach of the data-based Denoising Stage. Although more Dedicated MLP-based DAEs (one per denoised measurement) are required, their low complexity and good performance are crucial to make the choice. Lastly but not less important, this low complexity entails that the network will be able to denoise the measurements in less time than the other two methods, and therefore, no extra delays have to be taken into account (see Figure 8). This is also motivated by the training process where noise-corrupted measurements are referred to the ideal ones. In terms of the denoising behaviour, here it is observed that again, the worst performance if offered by the low-pass filter approach due to their implicit delay and low denoising accuracy. On the other hand, the best one corresponds to the Dedicated MLP-based DAEs which offer clean measurements practically identical to the ideal measurements. In other words, the measurements obtained when BSM1 sensors do not add noise and delays, i.e., when their ideal configuration is applied.

### 4.2. ANN-Based Delay Correction Performance

The ANN-based Delay Correction stage considers MLP networks whose main objective is to predict the difference between the cleaned measurement of the $S_{O,5}$ sensor and the $S_{O,5}$ concentration observed six minutes later. This amount of time is selected to assure that all the delays introduced by the non-ideal *A* sensors (1 min) as well as by the delays of the actuator (4 min) are corrected. In addition, some extra time is also included in this six minutes to correct the minimum delay introduced by the data-based Denoising process. In those cases where measurements of $B_{0}$ sensors are considered, the delay is nearly completely reduced since MLP networks will receive more information from type $A_{0}$ sensors. Thereby, they will be able to correct the delays of $S_{O,5}$ measurements even tough the delay introduced by $B_{0}$ sensors is bigger than the prediction time (six minutes).

As it has been stated previously, three different configurations of MLP networks have been considered accordingly to their input variables or measurements. Their training performance is shown in Table 3, where the training results are given in terms of the MSE and the $R^{2}$ coefficient as well as in terms of the training and test datasets. Here, a 75% of data has been considered as the training dataset, a 15% as the validation one and finally, the remaining 15% for testing purposes. The hyperparameters of the MLP networks have been obtained with the training and validation data. The final performance has been computed with data which is not considered in the training process, i.e., the test dataset.

As it is observed, all the configurations are offering a good performance since their *RMSE* values are below 0.03 and their $R^{2}$ coefficient is bigger than 0.93. However, there is a structure overcoming the other two. It corresponds to ANNconf3 configuration, which is able to improve the *RMSE* and $R^{2}$ of the ANNconf1 configuration in a 44.64% and a 4.50%, respectively. Besides, the improvement of ANNconf3 when compared to the ANNconf2 equals to a 34.16% in the case of the *RMSE* and a 2.25% in the case of the $R^{2}$.

Overfitting is not observed in the results of the Table 3 since an offset between the performance of the training and test datasets is not appreciated. This means that the three proposed configurations are not memorising the pairs of input and output data. Thus, the performance will not drop drastically if these nets are adopted as a delay correction method in similar scenarios.

**Figure 9:**
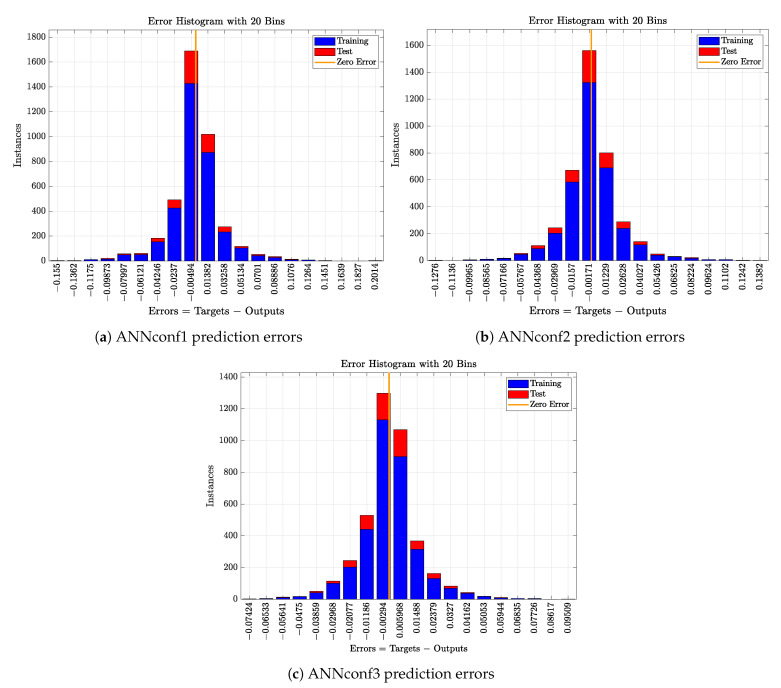
Distribution of prediction errors for the training and test datasets.

Finally, the distribution of the ANN prediction error, understood as the difference between the targets and the predicted values, has been analysed to determine if errors are biased towards a value or if they are centred to the zero error point (see Figure 9). It is observed that nearly all the errors are distributed around the zero error point, which means that predictions are correctly performed. In addition, nearly all the errors do not exceed an absolute error bigger than 0.06, less than 100 instances are above this error. As it happens with the data-based Denoising approach, this can be translated into the range of $S_{O,5}$ values where the clean and delay-corrected measurement will be placed. This range will equal to [1.94,2.06] if the maximum absolute error is considered as 0.06. This will entail that although there is a configuration performing better than the others, any of them can be considered in the ANN-based Delay Correction stage implementation.

### 4.3. Control Performance

After analysing the effects of the new proposed data-based Denoising stage as well as the ANN-based Delay correction process, we will compute the performance of the whole structure. The data-based Denoising stage has been implemented considering the Dedicated MLP-based DAEs and all the configurations proposed in the ANN-based Delay Correction stage. In addition, performance of the whole control structure (see Figure 3) will be computed considering fix and variable set-points. Variable set-points are considered due to the fact that most of the times the set-points considered in a control loop are either determined by another control strategy, or directly by certain parameters which vary along time. For instance, the considered set-points in [23,26,63] correspond to variable ones which have been determined by means of reinforcement learning methods, hierarchical control structures, or algorithms devoted to finding the optimal set-point to achieve the best WWTP effluent quality. In such a context, the fix set-point will equal to a $S_{O,5}$ concentration of 2 mg/L whilst the variable set-point will be determined following the hierarchical control presented in [63].

#### 4.3.1. Fix Set-Point Results

Results when a fix set-point is considered are shown in Table 4. It is clearly observed that the Default PI-based control strategy is the one yielding the worst performance. This is directly related to the noise effect introduced by the real sensors. On the other hand, the performance is improved in all terms when measurements are denoised and delay corrected in the data-based Denoising stage and in the ANN-based Delay correction stage, respectively. The effects of both processes are directly observed in the *IAE* and *ISE* control metrics. The lowest *IAE* improvement, a 32.81%, is yielded by the ANNconf1 configuration when stormy weather is considered.

In addition, it is important to highlight that if measurements are denoised and their delays corrected, the gain of the controller can be increased and therefore, an improvement in the control performance achieved. This principle was implemented in [36] and therefore, it has been considered in the implementation of this proposal.

In terms of the control strategies where denoised and delay-corrected measurements are considered, one can observed that the best performance is not always given by the same configuration. When dry and stormy weathers are simulated, the designed and scenario-optimised strategy in [36] is the one offering the best results. This shows that the moving average low-pass filters and the PI controller have been exhaustively designed to offer such a good behaviour. Notwithstanding, our proposals do not differ too much from the best results. When dry weather is considered, the ANNconf2 configuration shows *IAE* and *ISE* values which are very close to the ones offered in [36]: they are only degraded a 4.38% and a 6.66%. This is also corroborated with the other metrics, the mean absolute error is degraded 0.005 units, the variance of the error differs in $8\times 10^{-4}$ units and the maximum error in 0.039 units. The same is observed when the stormy weather is considered. The *IAE* and *ISE* yielded by the ANNconf3 are degraded a 7.4% and a 16.67% respectively. In absolute values, *IAE* is degraded from 0.300 to 0.324 whereas *ISE* is increased from 0.022 to 0.024. The mean(|e|), the max(|e|) and the var(|e|) metrics differ 0.002, 0.143 and 0.0003 units, respectively.

When rainy weather is considered, results show that all the proposed configurations (ANN-based Delay Correction ones) are able to improve the best performance shown in [36]. Now, the best improvements are offered by the ANNconf1 and they correspond to a 14.57% in terms of the *IAE* and a 11.54% in terms of the *ISE*. The mean(|e|) and the var(|e|) are improved $7\times 10^{-3}$ and $2\times 10^{-4}$ units. However, the max(|e|) is increased from 0.180 to 0.290. These improvements are related to the abilities of the ANNs considered in the data-based Denoising and ANN-based Delay Correction approaches. ANNs are able to better model non-linear behaviours and variations. In such a fashion, rainy weather is the one offering a bigger variation in the influent values since two long episodes of rain are produced between days 8 and 10 (see Figure 7a).

**Figure 10:**
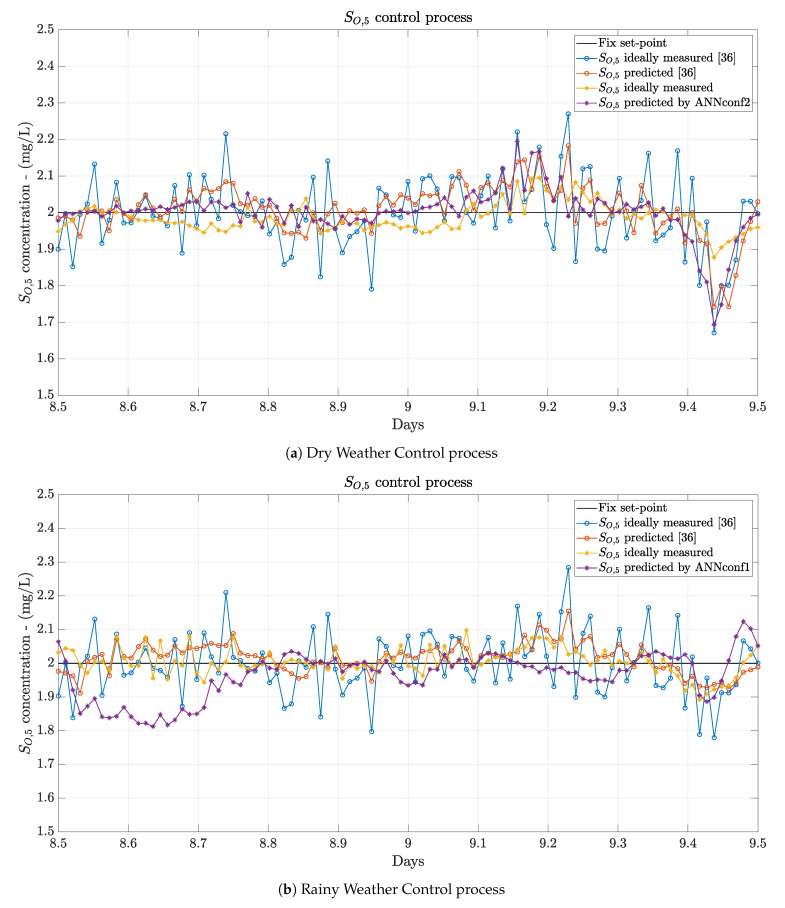

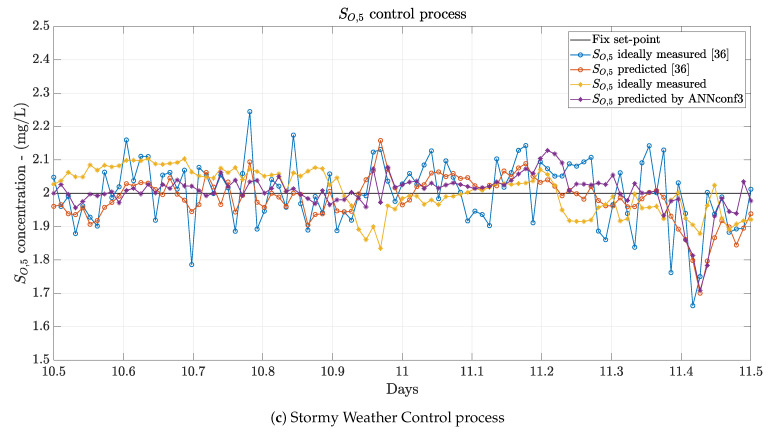
Control processes for the different weather profiles. Only the days where the effects of the weather events are predominant are shown.

Finally, in Figure 10 the performance of ANNconf2, ANNconf1 and ANNconf3 for the Dry, Rainy and Stormy weathers are compared to the best performance in [36]. As it is observed, all the structures are offering a good control performance since they are able to maintain the $S_{O,5}$ at the desired level or very close values most of the time. Notice that changes in ANNconf1, ANNconf2 and ANNconf3 are produced by the daily variations of the influent profile. In addition, one can observe that measurements have been properly denoised and also that effects of delays are not present between the ideal $S_{O,5}$ and the predicted one.

As a conclusion, results corroborate that the data-based Denoising and the ANN-based Delay Correction approaches proposed here can be adopted to change the methodologies designed and optimised towards the WWTP scenarios. Even though data-based structures are not always offering the best performance, it is worth to decrease a little bit their accuracy at expense of increasing their scalability, decreasing the design complexity and easing the implementation of the whole solution.

#### 4.3.2. Variable Set-Point Results

Variable set-points are the ones being tracked mostly since most of the control strategies consider set-points determined by other control strategy under a hierarchical control structure, or directly by parameters of the industrial plant which vary along time. In such a context, results of the whole system performance when a variable set-point is considered are shown in Table 5. The same effects as in the fix set-point results are observed between the Default PI and the other control structures. However, the differences are not so big due to the fact that effects of noise are lower when variable set-points are considered [36]. Now, the best improvement in terms of the *IAE*, a 41.05%, is offered by the ANNconf2 configuration when it is compared to the Default PI structure and the dry weather is simulated.

When the performance of the proposed approaches is compared to the structure presented in [36], the improvement is reduced, however, results are still better. The structure performing better in the case of the dry weather is the ANNconf2, which is able to offer *IAE* and *ISE* values equal to 0.336 and 0.035, respectively. This entails an improvement with respect to [36] equivalent to a 13.84% in terms of the *IAE* and a 7.89% in terms of the *ISE*. These results are complemented with the mean(|e|), the max(|e|) and the var(|e|). The mean(|e|) is now 0.049 units lower than before, the max(|e|) is increased from 0.320 to 0.445, but its var(|e|) is reduced from 0.0052 to 0.0049. In terms of the rainy and stormy weather, the structure offering the best performance corresponds to the ANNconf3. This makes sense since this structure is the one considering not only the $T_{SS,in}$ concentration, but also the $S_{NO,4}$ one. Aforementioned, these two concentrations are two of the most affected ones when weather variations like stormy and rainy events are produced (see Figure 7). For instance, when rainy weather is considered, this structure is able to improve the *IAE* and *ISE* metrics from 0.482 to 0.364 and from 0.056 to 0.032, respectively. Thus, the lower the *IAE* and *ISE* values, the better the control performance. When stormy weather is considered, the *IAE* and the *ISE* varies from 0.428 to 0.422 and from 0.046 to 0.040, respectively. The enhancement is reduced in this case since the variations of stormy weathers are not maintained in time like the rainy ones. Instead, they consist in high variations during a very short period of time. The rest of the time similar variations to the dry profile ones are observed.

**Figure 11:**
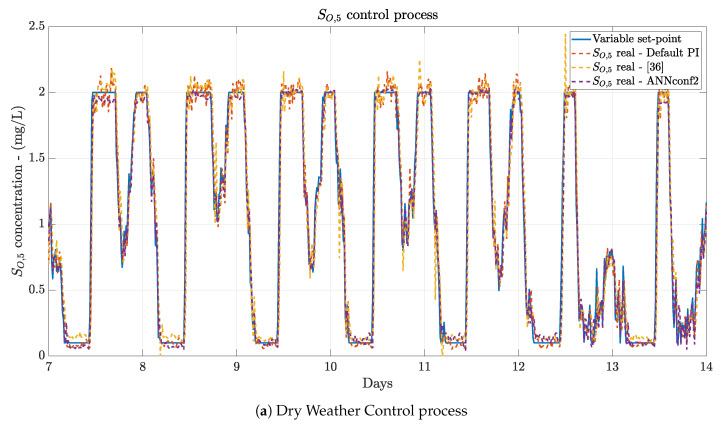

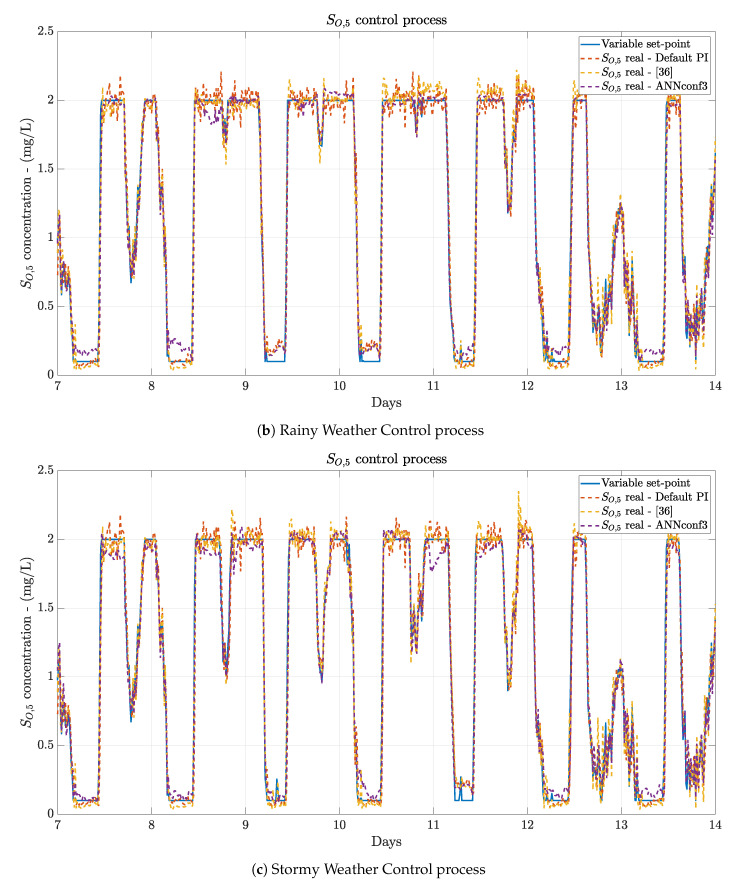
Control processes for the different weather profiles. Results are shown from day 7 to 14, the days established in the BSM1 simulation protocol.

Figure 11 shows the control process performed by the data-based Denoising stage when Dedicated MLP-based DAEs are considered and when the ANN-based Delay Correction Structure considers the best ANN configuration, i.e., the ANNconf2 for a dry weather and the ANNconf3 for the rainy and stormy weathers. Results of Default PI and [36] are also shown.

As it is observed, the proposed approaches are the ones performing better. When dry and rainy weathers are considered, the real $S_{O,5}$ concentrations obtained with the ANNconf2 and ANNconf3 are the ones closer to the variable set-point, i.e., the ones showing less oscillations as well as the ones showing no delays. Notice that there are some points where the $S_{O,5}$ obtained is not as reliable as it should be (see the lowest values of the variable set-point in Figure 11c). This effect is also observed when the stormy weather is considered. However, this is countered with the highest values of the set-point, where our approaches are making the point.

As a summary, our proposed approaches are offering the best performance when a variable set-point is considered. They are able to show the lowest errors at the same time they do not show oscillations when the set-point is maintained at constant values. It is true that there are some points where the obtained $S_{O,5}$ is not as close to the variable set-point as it should be. However, they are data-based approaches able to overcome the results of methodologies designed and optimised towards the WWTP scenario. This fact entails that these data-based methodologies allow a higher scalability, a lower design complexity and an easier implementation at the expense of losing some accuracy.

## 5. Conclusions

This work is focused on the implementation of two data-based methodologies to denoise and correct the delays introduced by sensors deployed over an industrial plant and therefore, improve its control behaviour. Here, the proposed methodologies are deployed and tested over the dissolved oxygen control loop of BSM1, a digital framework of a WWTP facility. Nevertheless, these methodologies are not exclusively to WWTPs. Their behaviour and results can be extrapolated to other industrial environments.

The two proposed methodologies consist in the data-based Denoising and the ANN-based Delay Correction stage. The former will be mainly focused on the denoising process of the measurements considered in the control process since the more precise the measurements, the better the control performance. In that sense, two ANN-based denoising approaches have been considered. The first one corresponds to a MLP-based DAE whose objective is to generate a clean version of the input measurements. The second approach consists in the Dedicated MLP-based DAEs which differ from the MLP-based DAE in the number of outputs. They estimate a unique clean output instead of multiple ones. As a consequence, as many Dedicated MLP-based DAEs as inputs have to be implemented. Both approaches have been compared with a classical denoising method, a low-pass filter, and they show that the best methodology consists in the Dedicated MLP-based DAEs. They offer an average *RMSE*, *MAE*, *MAPE* and $R^{2}$ metrics equal to 0.033, 0.025, 1.27% and 0.998, respectively. Not only this, the MLP-based DAE *RMSE* and *MAE* values have been improved a 64.89% and a 65.27%, respectively, when the Dedicated MLP-based DAEs are adopted. The second approach corresponds to the ANN-based Delay Correction stage, whose main objective is to correct the delays introduced by the different sensors and actuators. Simple MLP networks with three different configurations of input data have been considered to carry out this process. Results have shown a similar performance among the different configurations. Two of them offer *RMSE* values between 0.0287 and 0.0235 whereas the remaining one yields a *RMSE* equal to 0.0154, which is the lowest value. The novel point here is that these MLP nets have been trained with data gathered form an open loop configuration. In that manner, the effects of the controllers involved in the control approach are not considered and as a consequence, the delay correction is decoupled from the controller topology.

The whole system performance is computed to determine the improvement with respect to other approaches. Here, a PI controller has been considered as the main control tool in two types of control scenarios, one with a fix set-point and other where a variable set-point is adopted. Results show two evidences: (i) the whole system performance is improved in some cases when the fixed set-point is considered whereas (ii) it is always improved when a variable set-point is simulated. In the case of the fix set-point, it is observed that our approach is performing better only when rainy weathers are simulated. Nevertheless, the performance offered by our approaches are very close to the best ones when dry and stormy weathers are considered. For instance, when dry weather is simulated, the best *IAE* and *ISE* metrics yielded by similar approaches equal to 0.240 and 0.014, respectively. Our approach yields an *IAE* and *ISE* metrics equal to 0.251 and 0.015, respectively. In the case where a variable set-point is adopted, our proposal is the one overcoming all the other approaches indistinctively of the weather. The best improvement is again offered when the rainy weather is simulated. Our approach improves the *IAE* and *ISE* values around a 24.48% and a 42.86% with respect to approaches designed and optimised accordingly to the scenario where they are deployed.

In such a context, two points are observed. Our approach is able to improve the methodologies designed and optimised accordingly to the scenario when it is considered under certain weather circumstances. Similar performance is observed when fixed set-points are considered, while this approach performs better in those cases where a variable set-point, the most considered one, is adopted. However, the main point of our approach is that it is completely based on data and specially on ANNs. Therefore, only input and output measurements of the industrial plant are required. This entails similar or even a better performance with respect to methodologies and approaches designed and optimised to work in the given scenario at the same time we are decreasing the design complexity of the solution, easing its implementation process and also increasing its scalability. This is possible since neither mathematical models are required in the development of the delay correction methodologies, nor in the denoising process. All of them will be directly derived by the ANNs proposed in the data-based methodologies.

These points open a new horizon where the proposed system can be improved in different aspects. For instance, the controller can be designed and implemented considering only data-driven methods such as ANNs or Reinforcement Learning techniques. This will entail a new paradigm in the control of industrial processes since these kind of solutions will rely uniquely on data obtained from industrial plants at the same time they could be treated as ad-hoc solutions controlling harsh environments. Another interesting point arising from the results is that the performance of the proposed approach should be corroborated in real environments before applying it, not only in real WWTP facilities, but also in other industrial scenarios.

## Figures and Tables

**Table 1:** Parameters of the sensors involved in this work. The saturation level corresponds to the minimum and maximum measurable values.

Parameters of the Sensors			
**Measurement**	**Class of Sensor**	**Saturation**	**Delay**
$S_{O,4}$	*A*	0–10	1 min
$S_{NO,4}$	$B_{0}$	0–20	10 min
$S_{NH,4}$	$B_{0}$	0–20	10 min
$T_{SS,4}$	*A*	0–10,000	1 min
$Q_{4}$	*A*	0–100,000	1 min
$T_{SS,in}$	*A*	0–1000	1 min
$S_{O,5}$	*A*	0–10	1 min

**Table 2:** Results of the different data-based Denoising approaches. *RMSE* and *MAE* are expressed in (mg/L) for all the measurements except the $Q_{4}$ which is given in (m${}^{3}$/day). *MAPE* is a percentage and $R^{2}$ is a dimensionless variable. Training time is given in seconds.

Data-Based Denoising Stage					
**Classical Filter Approach [36]**					
**Variable**	***RMSE***	***MAE***	***MAPE***	$R^{2}$	**Training Time**
$S_{NH,4}$	0.203	0.129	14.65	0.959	-
$T_{SS,4}$	0.350	0.273	1.22	0.877	-
$Q_{4}$	0.215	0.155	4.75	0.966	-
$T_{SS,in}$	0.339	0.223	6.64	0.909	-
$S_{O,5}$	0.194	0.148	17.87	0.962	-
**MLP-Based DAE**					
**Variable**	***RMSE***	***MAE***	***MAPE***	$R^{2}$	**Training Time**
$S_{O,4}$	0.059	0.044	1.94	0.996	113.53
$S_{NO,4}$	0.078	0.063	1.29	0.994	113.53
$S_{NH,4}$	0.111	0.079	11.14	0.988	113.53
$T_{SS,4}$	0.127	0.102	0.46	0.984	113.53
$Q_{4}$	0.165	0.133	4.04	0.973	113.53
$T_{SS,in}$	0.085	0.063	2.07	0.993	113.53
$S_{O,5}$	0.034	0.022	5.18	0.998	113.53
**Dedicated MLP-Based DAE**					
**Variable**	***RMSE***	***MAE***	***MAPE***	$R^{2}$	**Training Time**
$S_{O,4}$	0.027	0.021	0.73	0.999	38.40
$S_{NO,4}$	0.039	0.030	1.00	0.998	43.04
$S_{NH,4}$	0.025	0.019	2.72	0.999	48.77
$T_{SS,4}$	0.055	0.043	0.39	0.994	41.71
$Q_{4}$	0.029	0.023	0.96	0.999	42.70
$T_{SS,in}$	0.033	0.024	0.97	0.998	49.80
$S_{O,5}$	0.022	0.016	2.15	0.999	62.25

**Table 3:** ANN Training performance.

ANN Training Performance			
**ANN Configuration**	**Dataset**	***RMSE***	$R^{2}$
ANNconf1	Training	0.0287	0.9419
	Test	0.0289	0.9376
ANNconf2	Training	0.0235	0.9655
	Test	0.0243	0.9597
ANNconf3	Training	0.0154	0.9833
	Test	0.0160	0.9818

**Table 4:** Control performance when a fix set-point is considered. All the errors are measured in mg/L. Results of the best structure are in bold.

Control Performance—Fixed Set-Point						
**Weather**	**Evaluation Criteria**	**Default PI**	**Best Performance in [36]**	**ANNconf1**	**ANNconf2**	**ANNconf3**
	mean(|e|)	0.084	**0.034**	0.043	0.039	0.041
	*IAE*	0.590	**0.240**	0.294	0.251	0.284
Dry	*ISE*	0.084	**0.014**	0.020	0.015	0.019
	max(|e|)	0.400	**0.160**	0.305	0.199	0.248
	var(|e|)	0.0120	**0.0018**	0.0031	0.0026	0.0028
	mean(|e|)	0.079	0.050	**0.043**	0.048	0.049
	*IAE*	0.560	0.350	**0.299**	0.335	0.348
Rainy	*ISE*	0.075	0.026	**0.023**	0.025	0.042
	max(|e|)	0.380	0.180	**0.290**	0.236	0.357
	var(|e|)	0.0110	0.0036	**0.0034**	0.0034	0.0058
	mean(|e|)	0.081	**0.043**	0.055	0.056	0.045
	*IAE*	0.570	**0.300**	0.383	0.380	0.324
Stormy	*ISE*	0.079	**0.022**	0.036	0.032	0.024
	max(|e|)	0.380	**0.210**	0.275	0.258	0.353
	var(|e|)	0.0110	**0.0030**	0.0051	0.0049	0.0033

**Table 5:** Control performance when a variable set-point is considered. All the errors are measured in mg/L. Results of the best structure are in bold.

Control Performance—Variable Set-Point						
**Weather**	**Evaluation Criteria**	**Default PI**	**Best Performance in [36]**	**ANNconf1**	**ANNconf2**	**ANNconf3**
	mean(|e|)	0.083	0.056	0.054	**0.049**	0.057
	*IAE*	0.570	0.390	0.381	**0.336**	0.372
Dry	*ISE*	0.124	0.038	0.035	**0.035**	0.043
	max(|e|)	0.983	0.320	0.342	**0.445**	0.580
	var(|e|)	0.078	0.0052	0.0048	**0.0049**	0.0073
	mean(|e|)	0.082	0.063	0.062	0.055	**0.052**
	*IAE*	0.564	0.482	0.420	0.381	**0.364**
Rainy	*ISE*	0.101	0.056	0.042	0.034	**0.032**
	max(|e|)	0.709	0.300	0.332	0.342	**0.299**
	var(|e|)	0.015	0.0065	0.0060	0.0047	**0.0044**
	mean(|e|)	0.083	0.059	0.079	0.069	**0.061**
	*IAE*	0.575	0.428	0.512	0.473	**0.422**
Stormy	*ISE*	0.115	0.046	0.093	0.052	**0.040**
	max(|e|)	0.983	0.341	0.835	0.398	**0.256**
	var(|e|)	0.0172	0.0062	0.0142	0.0079	**0.0059**

## Data Availability

The data considered in this study correspond to the influent and effluent data generated and available in the same BSM1 framework defined in [3,29].

## Abbreviations

The following abbreviations are used in this manuscript:

ANN	Artificial Neural Network
ASM1	Activated Sludge Model N.1
ASM2	Activated Sludge Model N.2
ASM2d	Activated Sludge Model N.2d
ASM3	Activated Sludge Model N.3
AWGN	Additive White Gaussian Noise
$b_{n,x}$	Biases of the *x*th hidden layer
BSM1	Benchmark Simulation Model No. 1
BSM1-P	Benchmark Simulation Model No. 1 with Phosphorus processing
BSM2	Benchmark Simulation Model No. 2
COD	Chemical Oxygen Demand (mg/L)
DAE	Denoising Autoencoder
e[n]	Error between set-point and output signal
FLC	Fuzzy Logic Controllers
*IAE*	Integrated Absolute Error
*ISE*	Integrated Squared Error
IWA	International Water Association
$K_{La,x}$	Oxygen Transfer Coefficient of the *x*-th reactor tank (day${}^{-1}$)
LSTM	Long Short-Term Memory Cell
$m_{a}$	Delay introduced by the actuator
$m_{s}$	Delay introduced by the sensors
*MAE*	Mean Absolute Error
*MAPE*	Mean average percentage error
MLP	Multilayer Perceptron
MPC	Model Predictive Controller
MSE	Mean-squared error
$N_{2}$	Nitrogen gas
$NO_{2}$	Nitrogen Dioxide
PCA	Principal Component Analysis
PI	Proportional integral controller
PID	Proportional integral derivative controller
*Q*	Flow rate
$Q_{x}$	Flow rate of the *x*th reactor tank
$Q_{a}$	Internal recirculation flow rate
$Q_{r}$	External recirculation flow rate
$R^{2}$	Determination coefficient
ReLU	Rectified Linear Activation Function
*RMSE*	Root Mean Squared Error
$S_{NO,x}$	Nitrate-nitrogen concentration in the *x*-th reactor tank (mg/L)
$S_{NH,x}$	Ammonium concentration in the *x*-th reactor tank (mg/L)
$S_{O,x}$	Dissolved Oxygen concentration in the *x*-th reactor tank (mg/L)
SW	Sliding Window
TN	Total Nitrogen (mg/L)
$T_{SS,x}$	Total Suspended Solids in the *x*th reactor tank (mg/L)
*u*	Actuation signal
$W_{n,x}$	Weights of the *x*th hidden layer
WWTP	Wastewater Treatment Plant
x	Noise corrupted signals considered in the DAE denoising method
$x_{n}$	LSTM input data
*y*	Controlled signal
$\hat{y}$	Denoised measurement
$\hat{y}$	Vector of denoised measurements
